# Composite Sickles and Cereal Harvesting Methods at 23,000-Years-Old Ohalo II, Israel

**DOI:** 10.1371/journal.pone.0167151

**Published:** 2016-11-23

**Authors:** Iris Groman-Yaroslavski, Ehud Weiss, Dani Nadel

**Affiliations:** 1 The Use-Wear Analysis Laboratory, Zinman Institute of Archaeology, University of Haifa, 199 Aba Khoushy Ave., Mount Carmel, Haifa, 3498838, Israel; 2 Martin (Szusz) Department of Land of Israel Studies and Archaeology, The Institute of Archaeology, Bar-Ilan University, Ramat Gan, 52900, Israel; 3 Zinman Institute of Archaeology, University of Haifa, 199 Aba Khoushy Ave., Mount Carmel, Haifa, 3498838, Israel; New York State Museum, UNITED STATES

## Abstract

Use-wear analysis of five glossed flint blades found at Ohalo II, a 23,000-years-old fisher-hunter-gatherers’ camp on the shore of the Sea of Galilee, Northern Israel, provides the earliest evidence for the use of composite cereal harvesting tools. The wear traces indicate that tools were used for harvesting near-ripe semi-green wild cereals, shortly before grains are ripe and disperse naturally. The studied tools were not used intensively, and they reflect two harvesting modes: flint knives held by hand and inserts hafted in a handle. The finds shed new light on cereal harvesting techniques some 8,000 years before the Natufian and 12,000 years before the establishment of sedentary farming communities in the Near East. Furthermore, the new finds accord well with evidence for the earliest ever cereal cultivation at the site and the use of stone-made grinding implements.

## Introduction

The use of plant resources as food is evident from the earliest days of humankind [1]. Likely, these foods were gathered by bare hands or by using simple ad-hoc tools. By the end of the Pleistocene, the Natufians (ca. 15,500–11,600 Cal BP) of the Near East commonly used a composite harvesting tool, the sickle. These sickles were composed of a handle made of bone or wood, and flint blade(s) or bladelet(s) inserted in it, as found at Natufian sites such as el-Wad in Mount Carmel, northern Israel [2] and Wadi Hammeh 27 in Jordan [3]. The hafted Natufian blades/bladelets have typical sickle sheen (later identified through use-wear analysis, see below). Such glossed elements, commonly termed sickle blades in local literature, were found in small numbers in many Natufian sites, and in larger numbers in the proceeding Neolithic sites [4].

Pre-Natufian sickle handles have not been found yet, and reports of isolated blades with faint sickle sheen are also rare. We report here the presence of five blades with apparent sheen at Ohalo II, a 23,000-years-old fisher-hunter-gatherers' camp on the shore of the Sea of Galilee. Furthermore, use-wear evidence for cereal harvesting is combined with hafting indications on some of the blades. This is the earliest evidence for such a composite harvesting implement, a tool that became a hallmark of Early Neolithic tool kits some 12,000 years later.

The aims of this paper are to describe the five Ohalo II glossed blades and the results of their use-wear analysis, to shortly present our experimental harvesting project, and to discuss the implications for reconstructing cereal economy at Ohalo II.

### The earliest evidence of sickles

In the Levant, the earliest appearance of glossed blades in the archaeological record is during the Early Epipaleolithic period. Although rare, isolated flint blades with weak sheen were mentioned from Kebaran sites (ca. 23,000–18,000 cal BP) such as Ein Gev I to the east of the Sea of Galilee: "A few blades with luster (undoubtedly close but not identical to the Natufian sickle blades) were also found" [5]. However, only in the Natufian do such elements become relatively common. In these sites, the sheen is found on both blades and bladelets, and their frequencies in the tool kits are usually in the range of <1%–5% (e.g., the Late Natufian sites of Eynan layer1b: 0.3% in northern Israel [6]; el-Wad 1.8% in Mount Carmel, northern Israel [7]; Fazael IV 4.8% in the southern Jordan Valley [8]).

The Natufian glossed implements, and for that matter the Neolithic ones too, were commonly perceived as associated with cereal harvesting. Indeed, already in 1892 Spurrel studied glossed items under the microscope and concluded that the glossy appearance was produced by friction with cereal stems [9]. Since then glossed blades and bladelets are commonly catalogued as sickle blades in the typo-technological analyses of flint assemblages in the Levant, and often interpreted as an indication of one of the most important developments in human subsistence technology, namely, the cultivation and later the domestication of cereals [10,11].

In the case of cereal harvesting tools, the investigation of Levantine Natufian and Neolithic sickle blades by the application of the methodological framework of use-wear analysis during the 80's and 90's provided a platform for reconstructing incipient agriculture, including aspects related to field conditions, species of crops and harvesting methods [12–17]. The study of cereal gloss was also a platform to investigate wear formation processes [18–22], and recently cereal gloss on flints was analyzed by the application of laser confocal microscopy [23,24].

Yet, it is reasonable to assume that cereal grains were consumed by humans much earlier than the fluorescence of the Natufian culture. In the Levant, the earliest examples include cereal grains found in Middle Paleolithic Kebara Cave in Mount Carmel, northern Israel [25] and Amud Cave in the Lower Galilee, northern Israel [26]. Evidence of cereal starch entrapped in European Upper Palaeolithic grinding tools demonstrates their use for plant food processing [27–29].

### The site of Ohalo II

**Fig 1:**
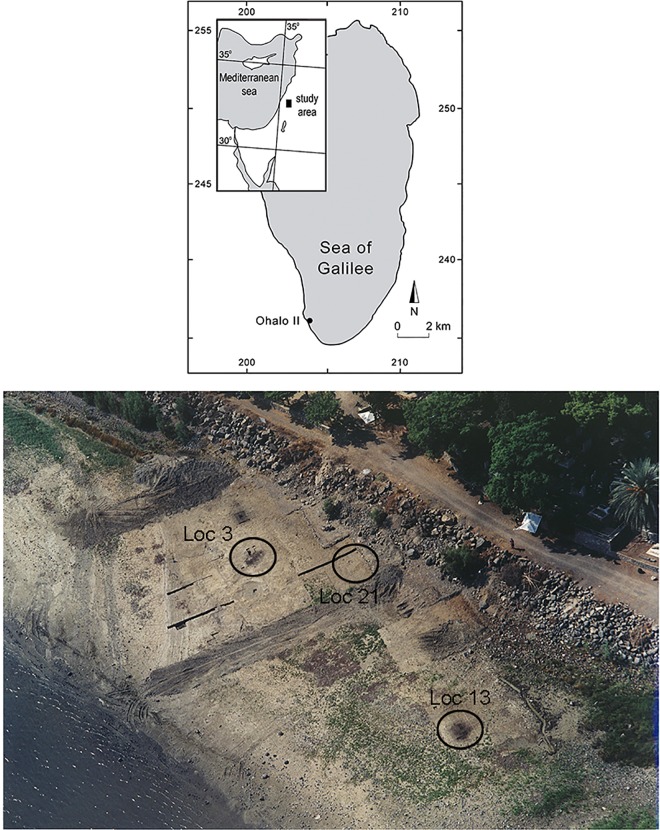
a) Location map of Ohalo II. b) An aerial view of the site; the three loci where the glossed blades were found are marked.

Ohalo II is located on the southwestern shore of the Sea of Galilee in Israel (Fig 1).

It was submerged shortly after it was abandoned and remained as such for millennia [30,31]. The site was dated by over 50 ^14^C dates of in situ remains, as well as of materials from pre-occupation and post-occupation layers [32–35]. Eleven loci were directly dated by 34 ^14^C readings, mostly ranging between 22,500–23,500 cal. B.P. [30,32,33,36]. Two of the loci with the items discussed here were directly dated: Locus 3 with 12 radiocarbon dates and Locus 13 with 3 radiocarbon dates.

The camp includes the remains of six brush huts and several clusters of hearths around them. In Brush Hut 1 the charred building materials of the walls were identified to the species level: thick branches of *Tamarix* sp. (Tamarisk), *Salix* sp. (Willow) and *Quercus ithaburensis* (Oak) were covered by smaller branches of plants such as *Atriplex/Seidlitzia* (Orach/Seidlitzia) and *Prosopis* sp. (Mesquite), as well as leaves and grasses [37]. Grass bedding encompassing charred bundles of *Puccinellia* cf. *convoluta* (Alkali-grass, a perennial grass) stems and leaves covered with a thin layer of compact clay was found on the bottom floor of Brush Hut 1 [36]. A grave of a male buried in a semi flexed position and a nearby stone circle were found near the brush huts [30].

A large faunal assemblage was found in all brush huts and fire places, including mammals, micro mammals, fish and birds [31,38,39]. A sample of ca. 160,000 charred and un-charred seeds and fruit remains comprising approximately 150 identified taxa was studied [40–44]. The presence of cereals in the plant assemblage is noteworthy. For example, on Floor II of Brush Hut 1 there were *ca*. 60,000 seeds and fruits, of which 606 are of *Hordeum spontaneum* (Wild barley) [45]. On Floor III there were *ca*. 55,000 specimens, of which 1,920 are from the same species [42].

Furthermore, evidence for grinding cereals on the floor of Brush Hut 1 was well-preserved. First, the unique distribution of cereal grains around a carefully set flat stone suggests the stone was used for food preparation [45]. Second, a thorough study of the stone, including sonication for the retrieval of microscopic remains, showed that the stone was indeed used for grinding wheat, barley and oats. The grinded cereals were supposedly used for making a baked product in an adjacent baking installation, which in turn increased the caloric intake of the inhabitants [46,47]. A use-wear analysis of the stone corroborates the previous conclusions [48]. Additional lower and upper grinding stones were studied, again reflecting the use of such implements in food processing at the site [48–50]. Furthermore, a recent botanical-ecological study showed that the Ohalo II people were cultivating cereals, probably on a small scale; this is by far the earliest example of its kind [51].

Thus, there is evidence at the site for cereal cultivation on the one hand, and for grinding and accordingly, consumption, on the other. Direct evidence for the relevant harvesting technology was missing. It is this gap that the current paper bridges by studying glossed flint implements, one of which was already briefly published [51].

## Materials and Methods

The methodology of use-wear analysis uses the principles of tribology to interpret wear patterns observed on archaeological tools [52]. The tribological characteristics of wear are used to infer the mode by which tools were used, including the action, the worked material and the manner of prehension or hafting.

Here we applied the combination of the low and high power approaches to observe and define the wear traces. At the first stage, the tools were observed under low magnification (a Nikon SMZ 745T stereoscope, magnifications 6.7-50x) in order to characterize edge removals and macro abrasions. At the second stage the tools were observed under high magnification (a metallurgical microscope Leica DM 1750M, magnifications 50-500x) in order to characterize striations, edge rounding and micro polishes. Wear traces were defined according to a list of attributes that are used in use-wear analysis protocols [53,54] and the functional aspects were reconstructed accordingly, including use motion, grip method and worked materials.

**Fig 2:**
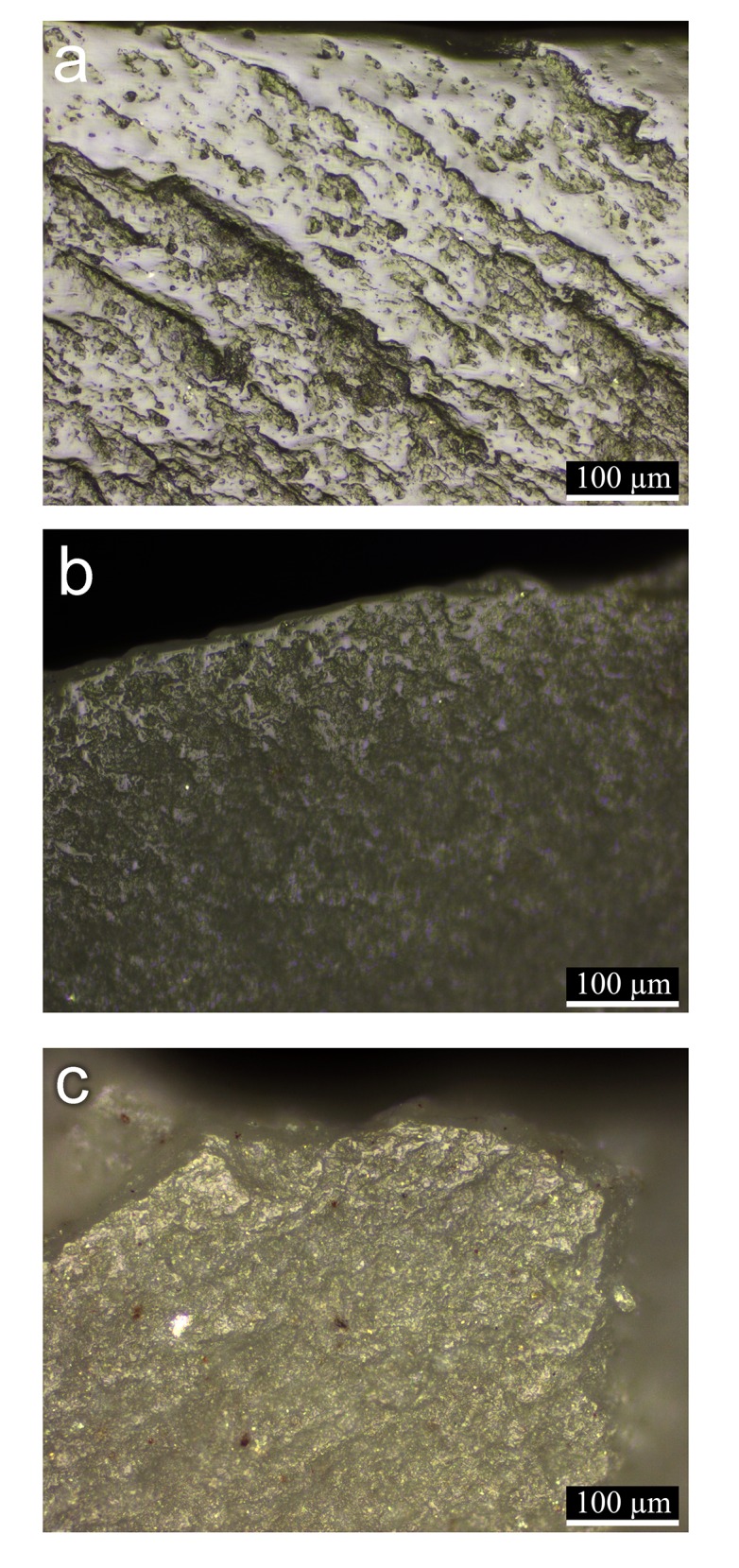
Micrographs showing nearly ripe semi-green cereal (wild oat) wear polishes observed on experimental tools: a: developed polish produced by sickle harvesting for 2 hours; b: polish developed to a lower degree produced by cutting with a blade held by hand for 1 hour; c: cereal prehension wear observed on the blade held by hand for cutting the cereals. Original magnification at all micrographs is x200.

In order to establish and confirm the functional reconstruction, the analysis results were compared to the reference collection available at the use-wear analysis laboratory at the Zinman Institute of Archaeology, University of Haifa (Fig 2 shows examples of experimental tools with cereal use and prehension wear relevant for discussing the Ohalo II glossed blades). So far, twelve cereal harvesting experiments were conducted in wild stands and domesticated fields in various locations including the slopes of Mount Carmel, northern Israel, and in the western Negev, southern Israel. These experiments did not involve endangered or protected species. Wild cereals were represented by wild oat (*Avena sterilis*) and barley (*Hordeum spontaneum* and *Hordeum bulbosum*); these were harvested in a semi-green condition, when the stems were in their full height and still green, and the grains almost ripe. Domesticated cereals were represented by wheat (*Triticum aestivum*); harvesting took place when the stems were dry and the grains were fully ripe.

Tools were used in the cereal harvesting experiments between 30 minutes and ten hours with the following main goals: a) to characterize use-wear polish of semi-green wild cereals and ripe domesticated cereals; b) to study wear formation rates by the application of sequential observations considering attributes such as angle of working edge and grain size of the flint; c) to assess the advantages and disadvantages of cereal harvesting using a composite sickle compared to a hand-held knife.

The harvesting experiments were conducted in a uni-directional cutting motion and the stems were cut at about 20 cm above ground, which was the convenient height to harvest standing and bending towards the stems.

The harvesting was done using composite sickles with three to five inserts made of flint of various grain size, inserted in a bone or a wooden handle, glued by bitumen or by a resin-bees wax mixture. In two experiments we used a blade held by hand. In one it was used for cutting stems, in the other for de-heading the ears by pressing the blade on the spikelets and pulling them off.

Another aspect that was examined in our analysis is the mode by which the tools were used, namely, the hafting and prehension arrangements. Our reference collection includes tools with traces produced by contact with hafts, by contact with bear hands or wrapped with hide; our inferences rely also on the extensive database provided by Rots [54]. By definition, prehension wear is mainly determined by the material being worked and it corresponds to the use-wear polish (for example, wood prehension wear or bone prehension wear). It is formed due to the contact with the residue of the worked material and it dominates the polish formation process. The distinction between prehension and hafting traces is based on the location of the traces over the tool. On the other hand, hafting traces are limited to a particular tool portion, where the haft is in contact with the flint element, and prehension and wrapping traces spread all over the area where the tool was held. Hafting traces are distinctive and include bright spots, rounded or abraded edges, specific scarring patterns and polish that correspond to the haft material [54].

### The glossed blades

**Fig 3:**
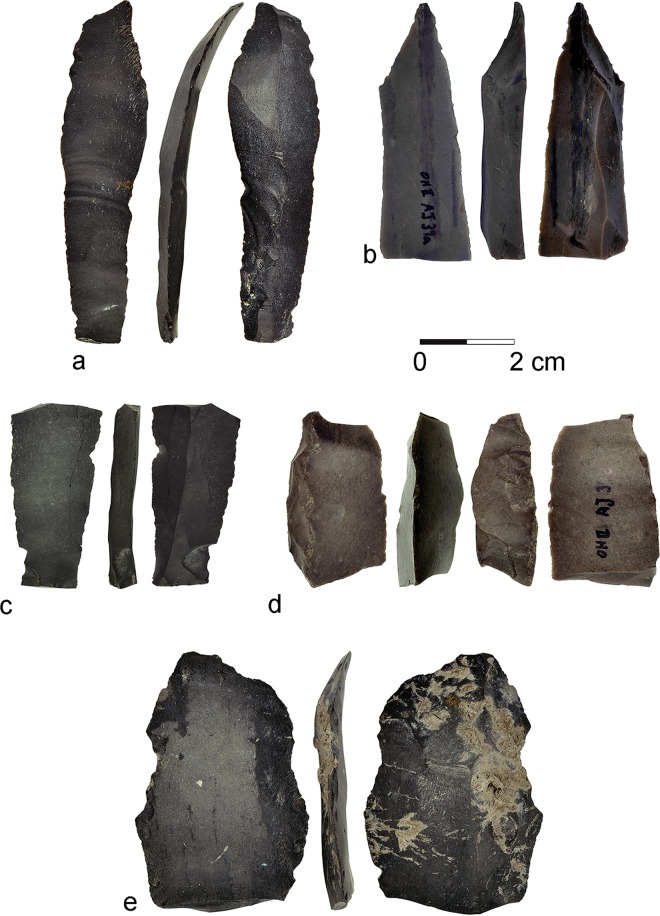
The five glossed blades from Ohalo II.

The Ohalo II flint assemblage is available at the Zinman Institute of Archaeology, University of Haifa. Five artifacts with macroscopically visible gloss were identified in the typo-technological analysis of the flint assemblage from Ohalo II (Fig 3).

Although the assemblage is large and over 100,000 specimens (excluding chunks and fragments < 1 cm) have been studied, the retouched tools comprise only 3.3% of the assemblage, and the glossed pieces are indeed rare (ca. 0.1% of the tools). Furthermore, the assemblage is bladelet oriented, and most cores and laminar elements reflect an industry focusing on small bladelets [33,34,55]. These form 41.5% of the assemblage, while blades form 11.4%, and most of them are small and similar to the bladelets. The retouched bladelets dominate the tool kit, as they form 60–70% of the tools in most loci. Other tools include retouched blades and flakes, and lower numbers of notches, burins and ad-hoc specimens. Interestingly, no gloss was identified on bladelets. Four of the glossed blades were found on radiometrically dated brush hut floors (two in each of Loci 3 and 13), and one in Locus 21 (at the edge of the excavated site).

The glossed blades vary in morphology and dimensions (Table 1). The blades were produced of local flint nodules commonly used at the site. A study of 300 complete blades from six loci clearly illustrates the general tendency to manufacture and use small blades: the average length in all six samples ranges between 32.1 mm and 39.1 mm (Table 2). Thus, all five specimens discussed here are much longer and, for that matter, are also wider and thicker than the average blade at the site. The glossed blades may have been produced on-site, as blade cores, large primary elements and large core trimming elements are present.

**Table 1:** Morphometric characteristics of the glossed blades from Ohalo II (dimensions in mm).

Artifact	Length	Width	Thickness	Cross section	Profile	Laterals
AJ39a (L13) Fig 4	58	15	6	Trapezoidal	Straight	Straight, converging distally
AL73c (L21) Fig 5	44	11	5	Triangular	Straight	Parallel
AJ39c (L13) Fig 6	40	20	12	Triangular	Straight	Parallel*
B86d (L3) Fig 7	78	17	5	Triangular	Curved	Straight, convex and converging distally
C86c (L3) Fig 8	65	37	6	Triangular	Curved	Irregular, converging distally*

*Broken at one end

**Table 2:** Average dimensions (mm) of complete blades from six loci, organized in descending order of length.

Locus		Length	Width	Thick.	N
3	AVG	39.1	14.1	4.5	92
	STD	12.5	3.7	2.0	
14	AVG	35.7	12.7	3.5	30
	STD	9.3	3.6	1.4	
18	AVG	35.7	13.2	3.9	19
	STD	11.3	4.2	1.9	
15	AVG	34.8	11.3	3.5	26
	STD	9.4	2.9	1.4	
19	AVG	34.7	12.8	3.5	75
	STD	8.7	3.7	1.9	
12	AVG	33.1	12.4	3.5	58
	STD	9.1	2.4	1.4	
Total	AVG	32.1	11.5	3.5	300
	STD	2.0	0.9	0.4	

## Use-Wear Analysis Results

**Fig 4:**
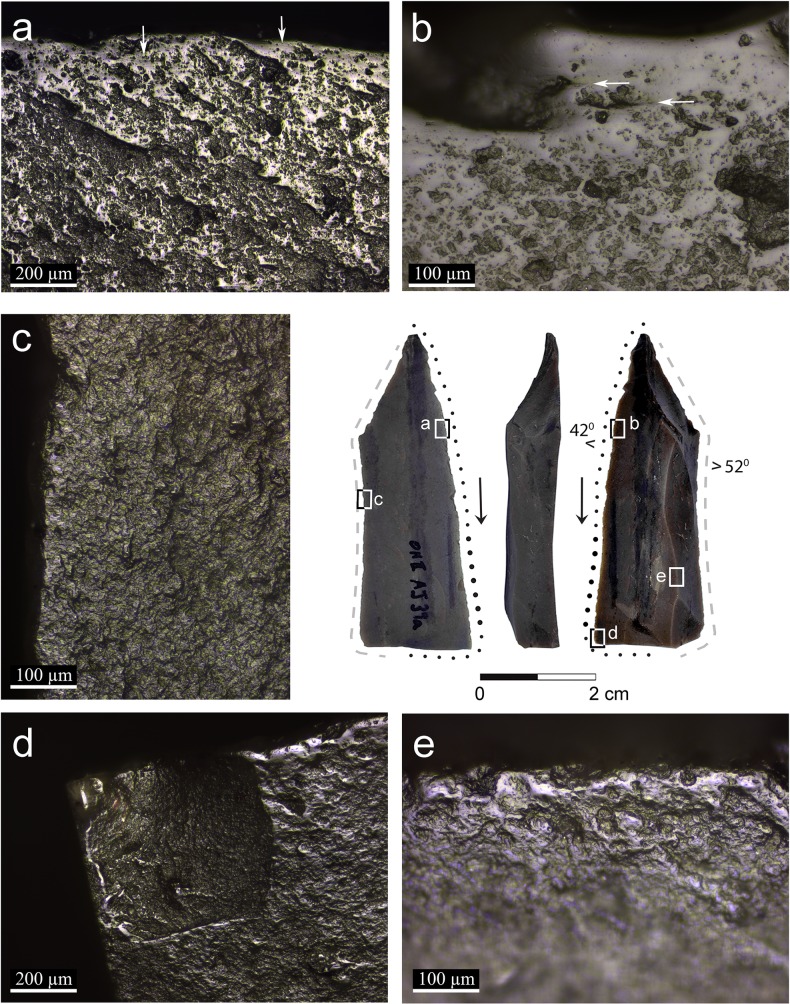
Artifact AJ39a with micrographs of the semi-ripe cereal use and prehension polish: a: general view showing the reticular distribution of the polish along the edge with arrows pointing at striations extending parallel to the axis of the blade indicating the longitudinal motion (x100); b: smooth and linked polish observed right on the edge fading into a reticular pattern towards the inner part of the blade with arrows pointing to comet shaped pits that indicate the uni-directional motion (x200); c: unmodified edge characterized by slight edge rounding and cereal prehension polish (x200); d: cereal use-wear polish observed on the broken edge indicating that the blade was used broken (x100); e: domed polish observed on the dorsal ridge indicating that it was exposed to the contact with the cereals (x200).

**Fig 5:**
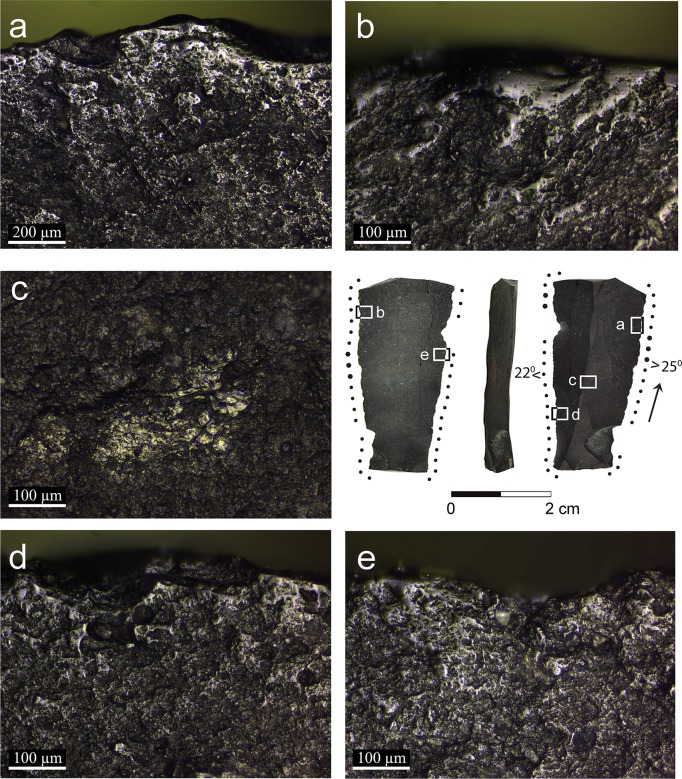
Artifact AL73c with micrographs of the semi-ripe cereal polish and hafting wear: a: polish developed to a low degree, brighter and reticular right on the edge (x100); b: smooth domed polish developed right on the edge (x200); c: a patch of flat bright spot observed on the dorsal ridge that might be an evidence to the contact with a haft (x200); d–e: polish on the opposite edge with identical characteristics indicating retooling (x200).

**Fig 6:**
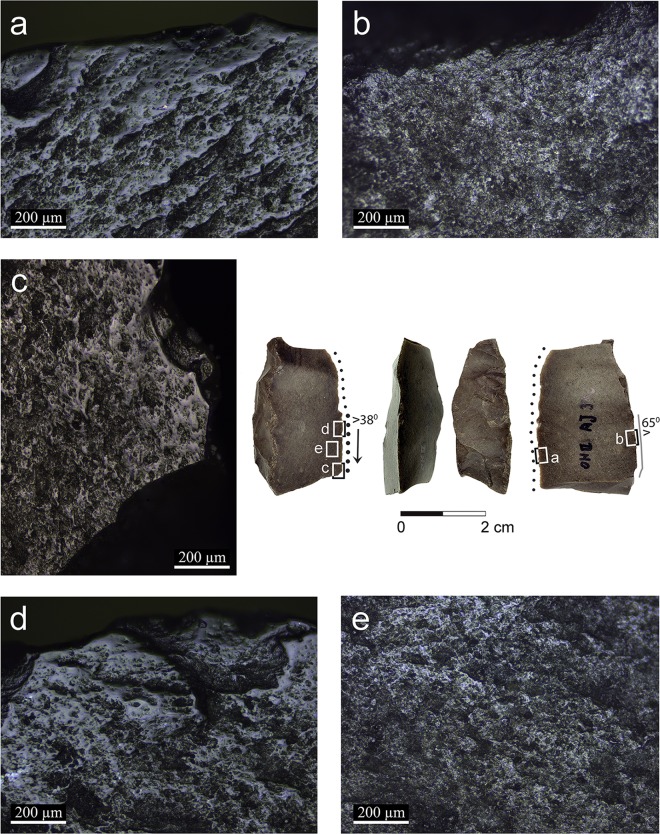
Artifact AJ39c with micrograph of the semi-ripe cereal polish and hafting wear: a: a reticular pattern with a relatively high degree of linkage between the polished surfaces (x100); b: streaks of dull rough polish associated with slight edge rounding interpreted to be produced by the contact with a haft (x100); c: reticular polish cut by the break indicating that the blade was broken after use (x100); d: linked and smooth polish developed on elevated points (x100); e: rough thin polish observed away from the developed linked polish fading gradually at the inner surface of the blade (x100).

**Fig 7:**
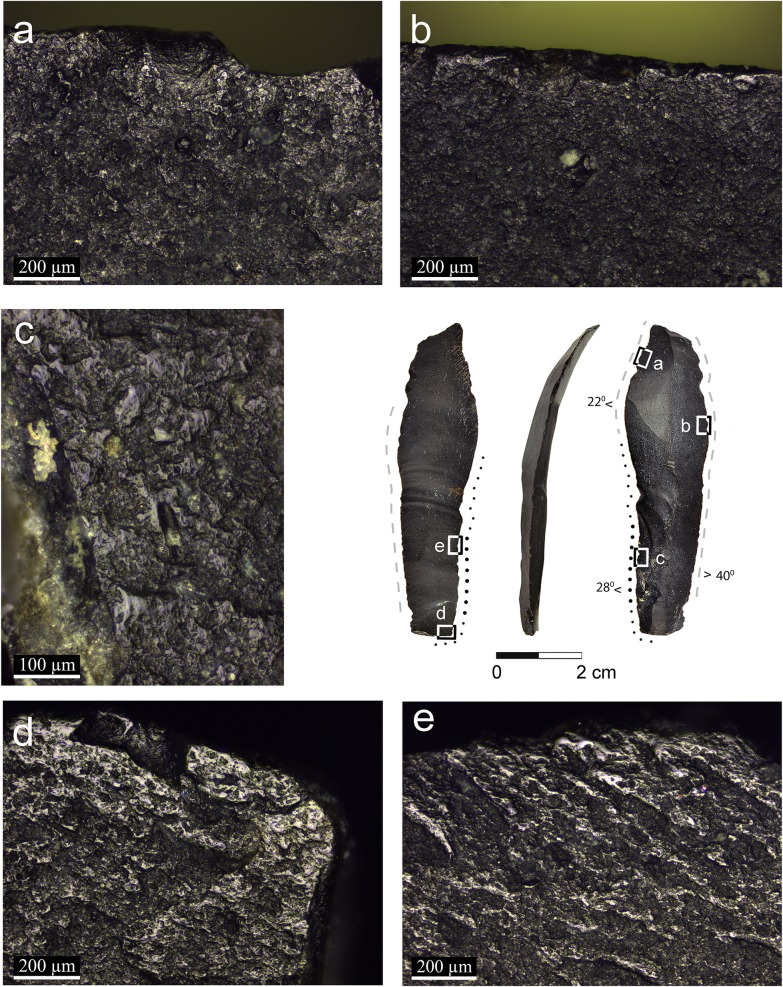
Artifacts B86d with micrographs of the semi-ripe cereal use and prehension wear: a–b: cereal prehension wear observed on the distal area of the blade indicating the location of the grip (x100); c: cereal use-wear polish developed to a low degree observed on the dorsal face (x200); d: polish observed on the proximal extremity indicating that this part was exposed to the contact with the cereals (x100); e:same wear pattern on the opposite ventral face (x100).

**Fig 8:**
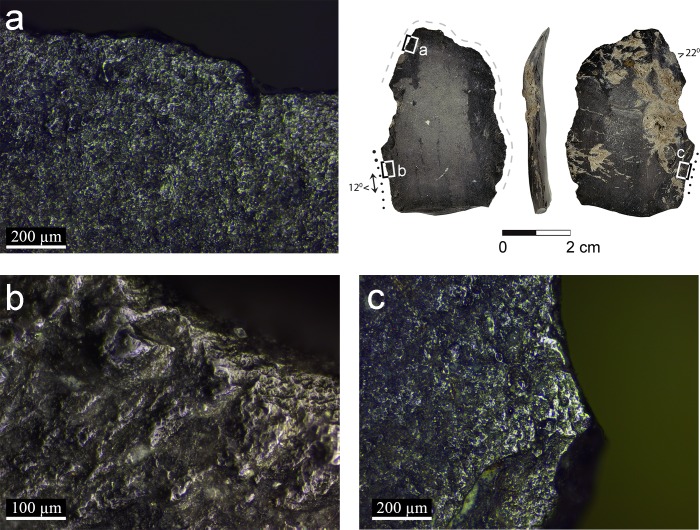
Artifact C86c with micrographs of the semi-ripe cereal use-wear polish and prehension wear: a: cereal prehension wear similar in its characteristics to the cereal use-wear polish, observed all along the right lateral edge, surpassing the distal part to the opposite left lateral indicating the area in contact with the palm (x100); b-c: semi-ripe cereal use-wear polish developed to a low degree, characterized by a reticular distribution and domed polish that developed on protruding surfaces of the flint, with linked polished surface observed right on the edge (x200, x100).

The results of the use-wear analysis of the glossed blades are presented by micrographs (Figs 4–8) comparing them to cereal use and prehension wear observed on the experimental tools (Fig 2).

In all cases, traces that could be attributed to post depositional surface modification (PDSM) are very light or entirely absent, indicating that the specimens are well preserved, providing an excellent opportunity to study their macroscopic and microscopic wear traces.

The glossy edges of the blades are unmodified and the sheen, evident to the naked eye, appears as a thin band along the edge. Under high magnification (usually x100-x200) the polish exhibits the characteristics of semi-ripe cereal use-wear polish, similar to the traces observed on the experimental tools (Fig 2A and 2B). It is smooth in texture, bright in appearance, undulating and domed in topography with a general distribution as a band along the edge. The presence of water in the semi-ripe stems is the most influential component in the use-wear formation process, causing a brighter and smoother polished surface. The hardness of the stem also plays an important role, as the harder ripe stems abrade the flint more intensively, creating the flatter, rougher and duller polished surface. The semi-ripe cereal use-wear polish is identified even where traces are developed only to a low degree, as the characteristics are evident on the polish located right on the edge of the tool or on the protruding surfaces (domes or reticulation). The linked polished surfaces observed right on the edge (less than 1 mm in width) is associated with a reticular pattern that characterizes the surfaces very close to it. Polish away from the edge is developed on protruding surfaces in accordance with the microtopography of the flint. It is also evident that the polish fades gradually away from the edge and along most of the length of the edge, which is a pattern that characterizes use-wear of cutting soft materials such as cereals.

In artifact AJ39a (Fig 4) use-wear polish is evident on the dorsal ridge (Fig 4E), indicating the wide surface exposed to contact with the stems. This is also characteristic of working with a soft plant such as herbaceous siliceous species, and not hard siliceous species such as reeds.

Two broken blades (artifacts AJ39a: Fig 4 and AL73c: Fig 5) exhibit polish on the proximal and distal broken edges (Fig 4D), indicating that they were used broken when the use-wear developed. On the other hand, the polish is cut by the breaks on the distal and proximal ends of artifact AJ39c (Fig 6C), indicating that the blade broke after being used in cereal harvesting.

Three blades exhibit traces interpreted to have been produced by prehension (artifacts AJ39a, B86d and C86c, Figs 4, 7 and 8), similar to the traces observed on the experimental tools (Fig 2C). The cereal prehension polish is interpreted to be produced by the contact between the tool and the palm with residue of the cereals during the cutting. It appears close to the edge, opposite the edge with the use-wear polish or adjacent to it, spreading on a large portion of the circumference of the tool's edge. The cereal prehension polish is similar in its characteristics to the cereal use-wear polish. It is observed on most of the perimeters of the three blades indicating the relatively free movement of the tool in the palm. The long and curved blade exhibits cereal prehension wear (artifact B86d, Fig 7) along the edges of the concave distal part of the tool and the proximal right edge (opposite the working edge, Fig 7A and 7B) and use-wear polish is observed along the straight proximal part of the left edge of the blade, including the area of the striking platform (Fig 7C–7E). According to this reconstruction it would seem that the holding of the tool might cause injury of the hand due to the sharpness of this portion. However, holding it feels different, as the tool aligns quite comfortably with the curve of the palm. The largest blade (artifact C86c, Fig 8) exhibits use-wear on a very short section of the edge (Fig 8B and 8C) and prehension wear was observed along most of the circumference of the tool adjacent to it. Another blade (artifact AJ39a, Fig 4) exhibits a long cutting edge (Fig 4A, 4B, 4D and 4E) opposite the edge with the cereal prehension wear (Fig 4C).

Two blades exhibit traces which were interpreted to have been produced by friction within the haft. One blade (artifact AL73c, Fig 5) exhibits bright spots along the edge opposite the edge with cereal use-wear polish (Fig 5C) and the other (artifact AJ39c, Fig 6) exhibits edge rounding associated with rough, dull polish and streaks of polish (Fig 6B). Compared to the blades interpreted to be held by hand, the hafting traces are restricted to the edge opposite the edge with use-wear.

Striations are extremely rare for all the five tools. They are detected on linked polished surfaces, under high magnification (x200) and can be observed only in specific angles (Fig 4A). They are long, narrow and shallow, extending parallel to the edge of the tool, indicating the longitudinal motion of the tool, perpendicular to the stems. Comet-shaped pits that indicate the uni-directional pulling motion of a tool (especially those hafted) were detected occasionally (Fig 4B). Based on our experiments, comet-shaped pits are rarely observed with polish that was developed to a low degree (for example, tools which were used for a short time of 30 minutes to 1 hour).

## Discussion

The glossed blades from Ohalo II are the earliest evidence for the use of flint inserts as part of composite cereal harvesting tools, appearing more regularly only in Natufian sites, some 8,000 years later. Our use-wear analysis results provide new insights pertaining to past technologies and subsistence patterns. Within the Ohalo II flint assemblage, the five glossed specimens are outstanding in their morphometric characteristics, as they are much larger than the average blades at the site. They were not a regular product of the local reduction sequence, mostly aimed at the manufacturing of bladelets and small blades, indicating the selection of large rare flint artifacts for harvesting.

The use-wear analysis shows that the Ohalo II glossed blades were used for cutting cereal stems at a near ripe semi-green condition. This was determined by comparison to our experimental tools where polish of identical characteristics was observed.

Cereal grains found at Ohalo II are of wild species [40,42,45,51] and harvesting them semi-ripe indicates that the Ohalo II people already knew that harvesting the ripe cereals with a tool would result in shattering off the grains and in heavy loss of potential food [56,57]. Using a sickle also enhances the harvest event in terms of speed and precision. One of the outcomes of such a method was larger quantities of cereal stems of regular length, available for use at the site. Evidence for cereal processing at the site is clearest in Brush Hut 1, where a grinding slab was firmly set on the floor; microscopic wild cereal starch grains were extracted from its surface, and a patterned distribution of cereal seeds was found around it [45–47].

The use of composite sickles at Ohalo II, inferred by the presence of typical cutting and hafting wear (Figs 5 and 6), is the earliest ever documented. It implies preplanned harvesting endeavors, as specific tools and materials were prepared in advance, including the handle, flint inserts and adhesives. Evidence for the preparation of composite hunting tools, i.e. projectiles with several barbs, is also present at the site [58]. The distribution of the traces on the glossed implements indicates that blades were placed relatively protruding and parallel to the axis of the haft. It is intriguing that no adhesive residues were found on the blades because the site is well preserved, and some such residues were found even on small retouched bladelets [58]. One possible explanation is that there was no use of mastic and the blades were inserted by pressure into the socket of the haft. We could not reconstruct the material from which the hafts were made, although wood implements have been preserved at the site [59]. Sickle hafts found in Natufian sites such as Kebara cave [60], el-Wad cave [2,61], Hayonim cave [62], Wadi Hammeh 27 [3,63] and Zawi Chemi Shanidar in northern Iraq [64] were all made of long bones and horns [65].

Cereal harvesting by hand-held blades has also been identified in our analysis (Figs 4, 7 and 8). By comparing the distribution of the prehension wear it is evident that tools were held according to their morphology, exploiting a preferred edge to be used as an active edge. Compared to composite sickles, these tools may represent a more opportunistic nature of cereal harvesting. Still, they are unique in morphology and dimensions compared to most of the blades at the site, indicating selection of appropriate items for harvesting.

The distribution pattern of the cereal use-wear polish (linked, smooth polish band, not wider than 1 mm right on the edge or very close to it, fading gradually in a reticular pattern) and its low intensity indicate short events of use. These differ substantially from Neolithic sickle blades in terms of sheen intensity and distribution on the specimen, commonly interpreted as reflecting multiple events of systematic harvesting [21]. Likely, the rarity of comet-shaped pits on the Ohalo II specimens also reflects low level of use. One blade interpreted to have been hafted (artifact AJ39c, Fig 6) was used more extensively than the others, but compared to our experimental tools it seems that each of the Ohalo II blades was used for no more than one or two harvesting days (or about 4 to 10 hours). One blade, showing two polished edges (artifact AL73c, Fig 5), likely represents an example of two events of utilization. However, both edges of this tool are still sharp and amenable for further work, therefore we cannot explain the reason of changing the working edge.

Large quantities of wild cereal grains were found at the site, including barley, wheat and oats [41–43,45,51,66], yet cereal harvesting and cutting tools are rare. The vast majority of the Ohalo II grains are smooth (*i*.*e*. harvested when ripe), and do not exhibit signs of puckering, which is characteristic of younger, unripe grains [67]. It is thus reasonable to suggest that a variety of collecting methods such as uprooting, hand stripping, beating into baskets or collecting from the ground [14,57,68–72] have been commonly used at Ohalo II.

The site was occupied year-round, as indicated by the remains of migratory birds and the timing of ripening of the identified plant remains [39,40,46]. The wide variety of plant species consumed and used at the site indicates the range of habitats exploited by the people and the wide dietary breadth exercised [40,44,47,73,74]. It would thus be reasonable to suggest that harvesting plant food in general, and cereal collecting in particular, incorporated several methods and tool types, according to season, distance from the site (e.g., carrying distance of tools and collected food), species, ripeness stage, and topographic location on the adjacent hill slopes.

Addressing the rarity of sickle inserts, this is also the case in some Natufian sites, where sickle blades are hardly present or missing altogether. In such cases, as well as for Ohalo II, several explanations can be forwarded:

1. Cereal harvesting using flint tools was a short duration task, therefore it is possible that polish hardly developed, and thus not visible to the naked eye. If this is indeed the case, it is possible that a systematic use-wear analysis of blades would reveal more tools.
2. Tools used in the field could have been discarded at the end of a day's work. This is especially plausible in the case of tools held by hand and in opportunistic harvesting. In the case of using composite sickles, the flint inserts could be lost in the field during the harvest, especially if no mastic was used to fix them. In two of our harvesting experiments we lost inserts although they were fixed with mastic made of resin, bees wax and lime powder, yet this mastic is not strong as bitumen.
3. Cereal stems may have been cut with flint tools only in rare cases; other techniques such as uprooting, basket collection and beating may have been the common way of harvesting.

Use-wear analysis of Natufian sickle blades has been applied to specimens from several sites in the Levant, including Abu Hureyra and Mureybet in Syria, Ain Mallaha, Hayonim Terrace, Hilazon Tachtit, Nahal Oren, El-Wad, Kebara, Hatoula, Jericho, Saflulim, in Israel and Iraq ed-Dubb in Jordan [14–16,20,75–82]. Looking at the published micrographs and the associated reconstructions, it seems that the studied implements have similar characteristics to those observed for the Ohalo II glossed blades, namely, the linked polish at the actual edge, fading gradually in a reticular pattern. These sickles were also interpreted to indicate short duration harvesting events. However, some of the Natufian sickle blades show highly developed polish, not observed on the Ohalo II implements. The higher numbers of sickle blades in some Natufian sites, combined with a more frequent evidence of developed cereal use-wear polish, can be used as indications of the intensification of cereal harvesting using composite tools. The larger numbers of Natufian grinding and pounding implements [83,84] accords well with this increase of cutting tools.

The distinction between semi-ripe and ripe cereal use-wear polish is an important aspect in the documentation of the evolutionary process of cereal cultivation leading later to domestication, as before domestication the harvest was most likely carried out at a semi-ripe stage, and for domesticated species it was at the ripe stage. The change in shattering characteristics and thus reaping stage and method of harvesting reflects a long process led by human selection [85,86]. The Ohalo II inhabitants were among the first to identify the ear-shattering problem and to find a technological solution. Unfortunately, due to poor preservation of organic remains in contemporaneous sites and even in most Natufian sites, we have no idea if the Ohalo II case was a short-lived isolated event that was re-invented thousands of years later, or if it was the beginning of a long continuous evolutionary process of which many of its stages are missing [87,88].

At any rate, the Ohalo II inhabitants also used grinding technology [46,48,50], albeit on a small scale, as is the case in isolated Upper Paleolithic sites in Europe [28,29]. According to the Levantine archaeological record, both harvesting and grinding technologies appeared more-or-less at the same time, and became wide-spread and commonly used only eight millennia later. The scarce archaeological remains of both technologies thus suggest a limited reliance of pre-Natufian societies on cereals. The Natufians, with their large sedentary or semi-sedentary settlements and their new subsistence patterns, were the first to incorporate and develop these two technologies to enable cereal consumption on a new wide level; shortly after it became the hallmark of the Near Eastern Neolithic village.

The excellent and rare preservation at Ohalo II provides a unique combination of finds that include cultivated cereal grains of two species, an in situ grinding slab with cereal grains around it, microscopic cereal starch remains and grinding use-wear evidence on its upper side, as well as the use-wear evidence on flint blades used for cereal harvesting in a semi-ripe stage. It is this range of finds that indicates that the Ohalo II people were innovative, and even without knowing so were the first on a long, likely intermittent, bumpy road finally leading to the establishment of agriculture.
